# Physicians’ preferences and willingness to pay for artificial intelligence-based assistance tools: a discrete choice experiment among german radiologists

**DOI:** 10.1186/s12913-022-07769-x

**Published:** 2022-03-26

**Authors:** Philip von Wedel, Christian Hagist

**Affiliations:** Chair of Economic and Social Policy, WHU – Otto Beisheim School of Management, Burgplatz 2, 56179 Vallendar, Germany

**Keywords:** Artificial intelligence, Physician preferences, Radiology, Discrete choice experiment, DCE, Willingness to pay

## Abstract

**Background:**

Artificial Intelligence (AI)-based assistance tools have the potential to improve the quality of healthcare when adopted by providers. This work attempts to elicit preferences and willingness to pay for these tools among German radiologists. The goal was to generate insights for tool providers and policymakers regarding the development and funding of ideally designed and priced tools. Ultimately, healthcare systems can only benefit from quality enhancing AI when provider adoption is considered.

**Methods:**

Since there is no established market for AI-based assistance tools in radiology yet, a discrete choice experiment was conducted. Respondents from the two major German professional radiology associations chose between hypothetical tools composed of five attributes and a no-choice option. The attributes included: provider, application, quality impact, time savings and price. A conditional logit model was estimated identifying preferences for attribute levels, the no-choice option, and significant subject-related interaction effects.

**Results:**

114 respondents were included for analysis of which 46% were already using an AI-based assistance tool. Average adoption probability for an AI-based tool was 81% (95% CI 77.1% − 84.4%). Radiologists preferred a tool that assists in routine diagnostics performing at above-radiologist-level quality and saves 50% in diagnostics time at a price-point of €3 per study. The provider is not a significant factor in the decisions. Time savings were considered more important than quality improvements (i.e., detecting more anomalies).

**Conclusions:**

Radiologists are overall willing to invest in AI-based assistance tools. Development, funding, and research regarding these tools should, however, consider providers’ preferences for features of immediate everyday and economic relevance like time savings to optimize adoption.

**Supplementary Information:**

The online version contains supplementary material available at 10.1186/s12913-022-07769-x.

## Background

### Artificial Intelligence and doctors with patterns

After the general foundations of AI research were defined in the early 1960s, studies on intelligent computer systems supporting physicians in treatment decisions already followed in the early seventies [1, 2]. Recent advances in computing power resulted in a revival of AI being more capable than ever thanks to techniques like Machine Learning (ML) or Deep Learning (DL). Today, ML and DL tools identify abnormalities in chest radiographs, scan electrocardiograms for myocardial infarction or detect hip fractures and breast cancer from imaging data at or above radiologist-level performance [3–6]. A current example is the application of AI to Computed Tomography (CT) and Magnetic Resonance Imaging (MRI) scans to facilitate diagnosis of COVID-19 [7–9]. Besides these effects related to the quality of care, research also shows positive economic effects of AI in healthcare via efficiency or productivity improvements [10]. From these examples it becomes apparent, that the so-called doctors with patterns that include radiologists, dermatologists or cardiologists are currently impacted by AI the most [11, 12]. These specialties generate rich sets of structured data which are essential to the training and application of AI-based algorithms. Especially radiologists are confronted by media and research with ever-improving algorithms even achieving above-physician performance and, hence, with a supposedly uncertain future [5, 13, 14]. Indeed, these specialists seem to think rather conservatively of AI. A survey among European radiologists, for example, revealed that almost 50% expect an increased workload due to the deployment of AI-based tools and 55% claim that patients will not be happy with an AI-based report. However, the same radiologists also seem to accept that a future without AI cannot be expected with 50% planning to use or already using AI [15]. Pressure also comes from other stakeholders of the healthcare system. In a recent survey among German patients, 57% supported a mandatory second opinion if the AI-tool was to perform better than the physician [16]. It becomes apparent that many radiologists and other doctors with patterns are already or will soon be confronted with the choice of AI-based assistance tools.

### Understanding preferences & willingness to pay for AI

It appears surprising that research on preferences regarding AI-based tools particularly of doctors with patterns is limited. Additionally, no study in this field has yet analyzed the monetary value physicians attach to these solutions. As already described, AI can positively impact the quality and cost-efficiency of healthcare. However, for healthcare systems to realize this value, physicians need correctly designed and acceptably priced assistance tool options. This study attempts to fill this research gap by empirically analyzing German radiologists’ preferences and willingness to pay (WTP) for assistance tools powered by AI. It thereby answers the research question of whether radiologists are generally willing to invest, and if so, what designs and pricing of these tools is preferred. To achieve this goal, a discrete choice experiment (DCE) was conducted. Whereas the majority of DCE studies in healthcare involves eliciting preferences from patients, this study takes a twist and highlights providers as the key decision-makers for AI-based investments. It attempts to create transparency on whether radiologists’ preferences are in line with industry’s current development trajectory. Furthermore, provider preferences can also provide room for thought for policymakers when deciding on potential industry sponsorships or research grants. In the end, the potential positive impact of AI in healthcare can only be leveraged via sufficient acceptance by the stakeholders relying on it every day.

## Methods

### The Discrete Choice Experiment and research question fit

**Fig. 1:**
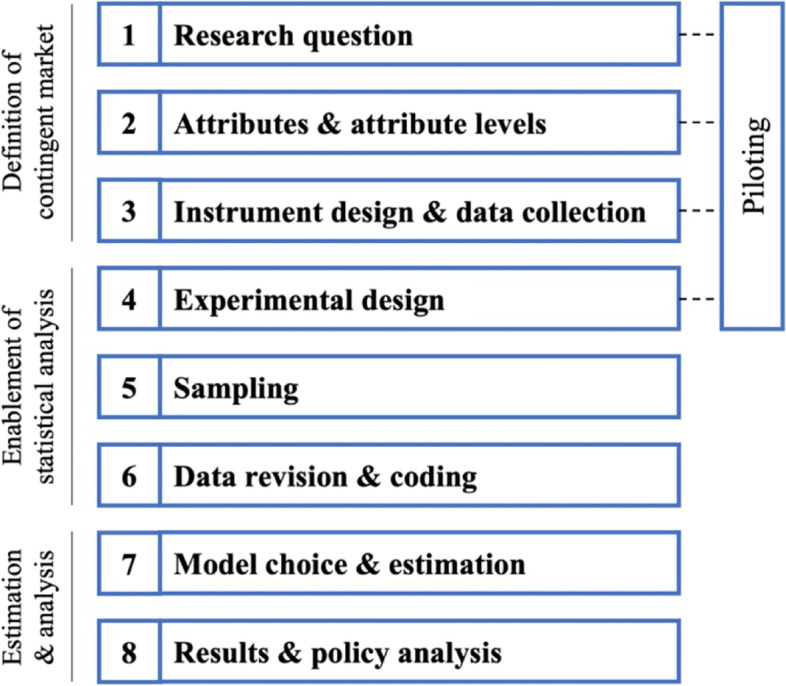
8-step process of conducting discrete choice experiments derived from literature

The DCE is considered an established tool in health economics research today [17–19]. DCEs represent a stated preference technique where participants face a hypothetical or contingent market scenario [20]. More precisely, Mangham, Hanson [21] describe the DCE as “a quantitative technique for eliciting individual preferences. It allows researchers to uncover how individuals value selected attributes of a program, product or service by asking them to state their choice over different hypothetical alternatives”. A price attribute can be included varying over different price levels to estimate WTP. Participants are then repeatedly asked to choose their preferred, or utility-maximizing, option [22]. These numerous decisions are econometrically analyzed to estimate the contribution of the single attributes to the overall utility. DCEs are regularly applied to examine patient preferences and WTP to derive recommendations on how to optimize care delivery from a patient perspective [23–25]. Provider preferences are significantly less studied; however they can also be an important factor in assuring optimal care delivery in specific cases. AI-based assistance tools are a perfect example here since these tools have the potential to positively impact healthcare, but providers are generally not obliged to adopt them. For several reasons, the DCE is a good methodological fit with the research question. Firstly, the researcher cannot observe an established market revealing preferences and WTP of radiologists due to limited information on the rather new market segment. The DCE resolves this situation by creating a choice scenario in a contingent yet realistic market. Secondly, this work does not solely focus on WTP, for which contingent valuation methods could be applied, but attempts to comprehensively analyze radiologists’ preferences for different compositions of AI-based assistance tools. DCEs allow for this analysis of multi-attribute goods and services. This study follows a comprehensive 8-step process of conducting a DCE derived from the established literature (see Fig. 1) [18, 20, 26–30]. The most pivotal steps of this process are covered in the following sections. All methods were carried out in accordance with relevant guidelines and regulations and in accordance with the 1964 Helsinki declaration and its later amendments.

### Identification of attributes and attribute levels

The identification of attributes and levels is considered a critical step when conducting a DCE [31]. The research question of this study requires the creation of a realistic contingent market for AI-based assistance tools for radiologists. To optimally mirror this market, an established four-step process including (1) raw data collection, (2) data reduction, (3) removing inappropriate attributes and (4) final wording was followed [32]. In the first stage, a long list of attributes and corresponding levels was developed based on online research on AI in radiology, information on commercially available AI-based solutions and recent scientific studies. Additionally, expert interviews with five radiologists from both hospitals (3) and outpatient practices (2) were conducted to complement the initial list resulting in a total of 7 attributes (see Supplementary Tables 1, Additional File 1). In the second and third stages, the experts were again asked to rank the attributes by perceived importance and perform sanity checks on attribute levels, ultimately defining a final shortlist. Experts clearly found 7 attributes to be too cognitively complex, especially keeping in mind the relatively high complexity of the application attribute. The aim was, hence, to include a maximum of 4 to 5 attributes. This process resulted in the exclusion of two initially developed attributes, namely “Automatic report creation” and “Automatic differential diagnosis proposal”. An automatic proposal of differential diagnosis implies the hypothetical assistance tool to execute the main application but to also simultaneously analyze all areas of the respective scan. Based on an AI-based analysis, the tool would then generate a proposal for differential diagnosis. Initial research, however, clearly showed that this kind of tool has not yet been introduced in practice and will likely need more time for development. Initial tests with the experts showed that tool options including this functionality were chosen less often. Experts indicated they were skeptical about the realizability of this functionality. This skepticism related to one attribute ultimately dominated the remaining attributes and levels. “Automatic report creation” was excluded since it was ranked less important in comparison with the remaining attributes. Furthermore, these attributes also provided minor policy relevance which is a key aspect to consider when choosing DCE attributes [19]. After wording was finalized, the final product option was composed of 5 attributes with a maximum of 3 varying levels each (see Table 1). As expected, the application attribute was discussed most in the interviews since it defines a key feature of the composed product. Together with the experts it was concluded that the application levels should ideally represent different archetypes of AI-based support. At the same time levels had to remain non-dominant, i.e., similarly interesting to respondents, thereby avoiding lexicographic behavior [20, 32]. The final levels of the application attribute represent three main categories of AI-based tools: routine diagnostics (level 1), process efficiency (2) and diagnostic screening (3). The option to include an application related to COVID-19 detection was discarded due to insufficient everyday relevance and to avoid time-dependent effects. For the routine diagnostics level, the aim was to choose an application that can be considered routine in the majority of radiology work environments. Hence, initially developed levels concerning brain and prostate scans involving rather delimited analyses were excluded. The application was then complemented by the attributes: provider, quality, and time savings. The experts agreed that impact on quality and time savings of the tools, as well as the trade-off between these were other key factors of interest. Furthermore, these attributes are also highly policy-relevant. Initially, the Quality attribute included the level “worse”. The interviews with experts, however, showed that tool options including this level overpowered all other attributes and options resulting in avoidance. Ultimately, the “worse” level was excluded to avoid dominant choice processes. Price was included as the fifth attribute to allow for WTP estimations. Price levels were chosen to be on a per study basis, compared to one-time investments or yearly payments, to mitigate a possible effect of differing study volumes in respondents’ practices or hospitals and to abstract from different economic endowments of the probands such as liquidity or credit constraints. The experts and information on pricing schemes of existing commercial solutions provided input on realistic price levels. As an additional sanity check, the final price levels were compared with the average reimbursement for MRI (€117.14), CT (€65.19), or mammographic screening (€62.07) scans included in the DCE for statutorily insured patients in Germany [33].

**Table 1:** DCE attributes and attribute levels for AI-based assistance tools in radiology

Attributes		Attribute levels		
		**1**	**2**	**3**
**1**	**Provider**	Modality manufacturer	RIS/PACS software provider	AI-software startup
**2**	**Application**	Automatic marking of lung lesions in thoracic CT and liver and kidney lesions in abdominal MRI *[Routine diagnostics]*	Reduction of scan times for 2D & 3D abdominal MRI sequences via AI-based data manipulation *[Process efficiency]*	Presorting of mammographic screening reports into “100% normal” (BI-RADS 1&2) and “suspicious” incl. automatic lesion marking *[Screening]*
**3**	**Quality**	Same: Detects anomalies you would detect, too	Better: Detects anomalies you would not detect even with long inspection	
	Displayed only for “Application” level 2:	Same: Same image quality	Better: Higher image quality	
**4**	**Time savings**	Low: Diagnostics process 10% faster	Medium: Diagnostics process 30% faster	High: Diagnostics process 50% faster
	Displayed only for “Application” level 2:	Low: MRI scan process 10% faster	Medium: MRI scan process 30% faster	High: MRI scan process 50% faster
**5**	**Price**	3€ per study	6€ per study	9€ per study

Note: Italic text in *[brackets]* indicates application archetype here but was not shown to respondents

### Creation of realistic and efficient instrument and experimental designs

**Fig. 2:**
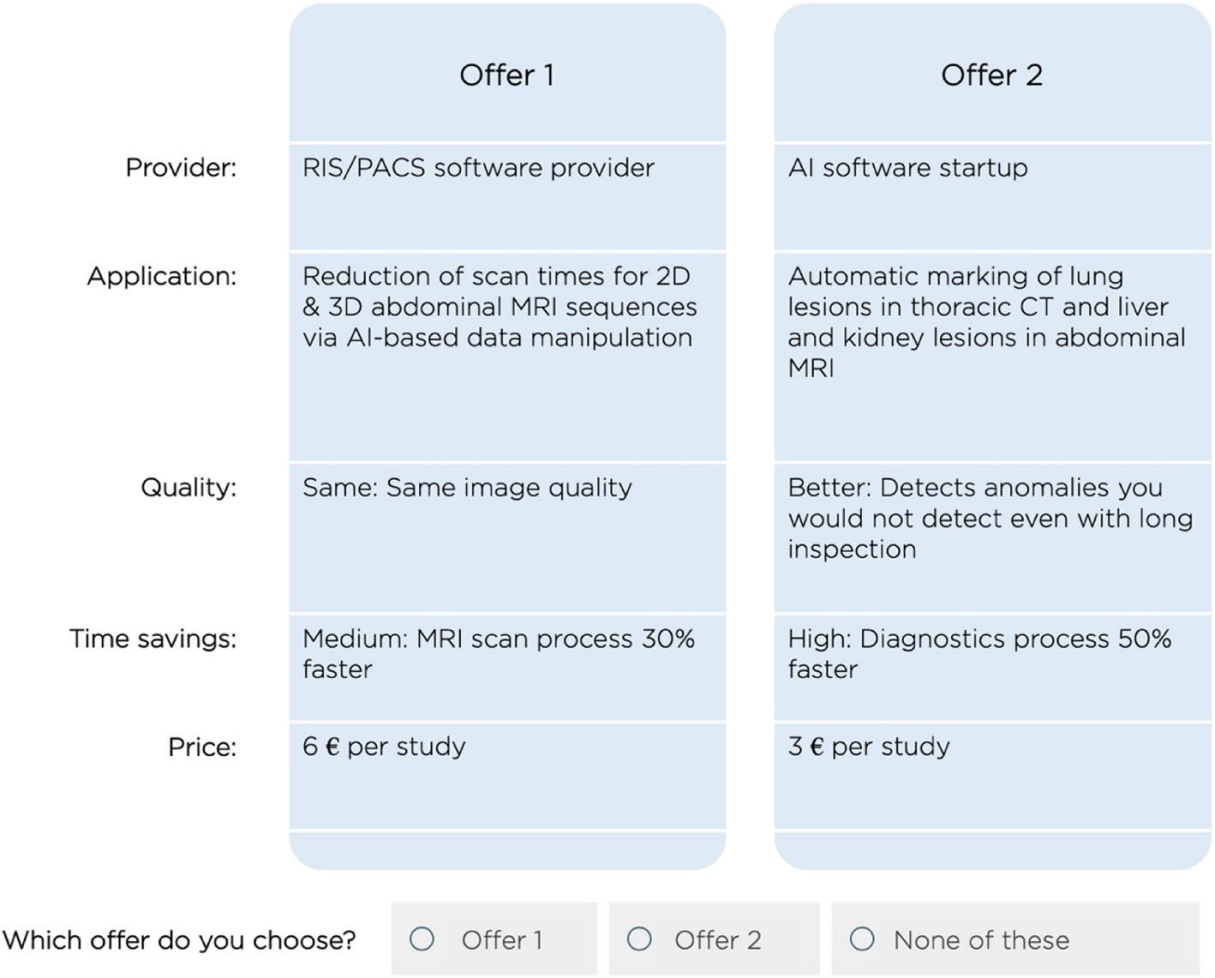
Example of choice set with two alternative offers and no-choice option

In this study, radiologists were confronted with a situation in which they were asked to choose between offers for AI-based assistance tools from different providers. As proposed by literature, respondents were also able to select a “no-choice” option to mirror a realistic decision process [34]. Respondents were asked to complete 10 choice sets consisting of 2 product variants each and the “no-choice” option (example in Fig. 2). As suggested by the literature, we added a detailed written-out explanation of the alternatives below each choice set to minimize choice inconsistencies [35]. The goal was to create a comprehensive yet simple instrument keeping scarce time of radiologists in mind. To achieve this, the instrument was piloted by the same experts the interviews were conducted with. Preceding the DCE, the first part of the instrument collected additional information keeping later statistical analysis of interaction effects in mind (see Additional File 2 for the complete instrument). Defining an efficient experimental design represents another key step in the setup of a DCE. A perfectly efficient design would consist of all possible combinations which is often not feasible. An established approach in design theory to circumvent this issue is the creation of fractional factorial designs that draw samples from all possible combinations [20]. To assure a statistically efficient way, researchers predominantly rely on the established measure of D-efficiency [18, 36]. For this study, a D-efficient design was generated utilizing the JMP 15 software by SAS. The resulting design with 92.35% D-efficiency is presented in Supplementary Tables 2, Additional File 1.

### Sampling approach

A sampling approach involves the definition of a sample frame, the setup of a sampling strategy and the determination of a minimum sample size via power analysis [37–39]. The sample frame was defined as fully trained radiologists and resident radiologists actively practicing radiology in Germany, hence excluding retired specialists. This results in a sample frame of 9,313 fully trained German radiologists in December 2020 [40]. Unfortunately, there is no reliable public information on the number of resident radiologists. Germany represents a robust study region since it can be considered as one of the largest uniform healthcare markets worldwide with very similar (statutory) reimbursement and treatment patterns applying to all providers and patients [41]. In terms of sampling strategy, the authors initiated a partnership with the two major professional associations of German radiologists. An online link to the survey including the DCE was circulated via established mailing lists to members of the “Berufsverband der Deutschen Radiologen e.V.” (BDR) and the “Deutsche Röntgengesellschaft” (DRG). To assess statistical power, the common rule of thumb by Orme [39] is applied resulting in a minimum sample size of $n>\frac{500*3}{10*2}=75$ to allow for the estimation of a reliable model.

### Model specification and analysis

A DCE attempts to mirror the process of utility maximization of the respondents. Following established literature, this utility ($U$) is composed of an observable part ($V$) and an unexplainable (random) component ($\epsilon$) representing unmeasured preference variation [20, 22]:

1 $$U=V+\varepsilon$$

The observable part is regularly described as an estimated linear-in-parameters function where the $\beta$s represent part-worth utilities or preference weights of the attribute levels $x$:

2 $$V={\beta}_1{x}_1+{\beta}_2{x}_2+\dots {\beta}_k{x}_k$$

The conditional logit model introduced by McFadden [22] has established itself as the workhorse for statistical estimation of the preference weights in choice experiments. We estimated fixed effects conditional logit models via the JMP 15 software. The software applies effects coding which can be detected when taking a closer look at the resulting parameter estimates. The no-choice option is represented as an additional dummy variable by JMP. Marginal Willingness to Pay (MWTP) for the single levels was calculated by dividing the estimated beta of the attribute level by the negative of the estimated price beta [42]. Relative attribute importance was calculated by dividing the range of marginal utilities of the respective attribute by the sum of all attributes’ ranges [43]. General choice or investment probability was calculated as.

3 $$P(1)=\frac{1}{1+\exp \left({\beta}_0\right)}$$

where ${\beta }_{0}$ represents the estimate of the no-choice dummy variable [20, 44]. To determine model fit, -2LogLikelihodd, the corrected Akaike Information Criterion (AICc) and Bayesian Information Criterion (BIC) were determined. A total of four MNL models were estimated incorporating several subject-related interactions. These were considered only when being statistically significant at 5% indicated by the p-value. The first MNL model, or simple model, represents main effects only without interactions. For this model, comprehensive utility metrics, MWTP and relative importance weights were additionally prepared.

## Results

### Sample size and characteristics

A total of 119 radiologists completed the survey between September 2020 and March 2021. Of those, four retired respondents and one respondent with a different department were excluded resulting in a final sample size of 114. For quality control, a minimum required time for the choice set completion of 15 s for the first and 5 s for the following choices was defined. All 114 respondents passed this test. Taking power analysis into account, our final sample size exceeded the calculated minimum required size of 75. Respondents were mostly male (78%), on average 51 years old (SD: 9.63) and predominantly working in an outpatient setting (74%) with mostly more than 13 years of fully trained years of work experience (68%) (see Table 2). Almost half of the respondents (46%) reported to use AI-based applications already today, which is in line with previous research [15]. The most utilized ones were applications that support or speed up the diagnostic process (42%). Only 35% indicated they were planning to invest in AI in the future with 46% being unsure about future investments (see Table 3). Interestingly, today’s users are more likely to also invest in further AI in the future. Non-users appear to be much more skeptical with the majority having no plans or being unsure (see Supplementary Fig. 1, Additional File 1 for a visualization). Besides this finding, users and non-users do not significantly differ in terms of other respondent characteristics.

**Table 2:** Sample characteristics

Sample characteristics *n* = 114		Absolute	% or (SD)
**Gender**	Male	89	78%
	Female	24	21%
	Diverse	1	1%
**Age**	Mean	51	(9.63)
	Min	27	
	Max	72	
**Job position**	Total outpatient	84	74%
	Employed in practice	18	16%
	Self-employed in practice	66	58%
	Total inpatient	29	25%
	Head physician in hospital	8	7%
	Consultant physician in hospital	16	14%
	Assistant physician in hospital	5	4%
	Employed in public authority	1	1%
**Specialization**	No specialization	66	58%
	Interventional radiology	2	2%
	Pediatric radiology	1	1%
	Mamma (breast) diagnostics	14	12%
	Musculoskeletal diagnostics	11	10%
	Neuroradiology	8	7%
	Oncological diagnostics	9	8%
	Other	3	3%
**Years of work experience as fully trained radiologist**	1–3	4	4%
	4–6	7	6%
	7–9	10	9%
	10–12	10	9%
	13+	78	68%
	Not fully trained	5	4%
**Average # of reports created per day**	Mean	43	(26.6)
	Min	0	
	Max	200	

Note: Rounded figures

**Table 3:** Sample exposure to AI

Sample exposure to AI *n* = 114		Absolute	%
**AI-based applications used today?**	Yes	53	46%
	No	57	50%
	Unsure	4	4%
**Type of AI-based applications used today** ^a^	Supporting/speeding up diagnosis	45	42%
	Prognosis of course of disease	4	4%
	Creation of reports	23	21%
	Improvements for image quality	16	15%
	Shortening scan processes	8	7%
	Replacing/reducing contrast agent usage	5	5%
	Practice/station management (e.g., claims processing. process optimization)	5	5%
	Other	2	2%
**Plans for future investments in AI-based applications?**	Yes	40	35%
	No	21	18%
	Unsure	53	46%

^a^Numbers do not add up to 53 users since respondents could choose multiple options; Note: Rounded figures

### DCE results

The estimation of the simple model yielded significant effects at a 1% significance level for all but the provider attribute. The significant attributes were, hence, relevant for respondents when deciding between the different AI-based assistance tools and can be considered to derive the preferences of the sample. The single utility parameter estimates, MWTP and importance weights are presented in Table 4. A detailed overview of model statistics can be found in Table 5 (MNL I). A summary of all parameter estimates and general answer to the research question yields the following: Radiologists prefer an AI-based assistance tool which supports in routine diagnostics with a better-than-radiologist quality and time savings of 50% at a price level of €3.00. A startup as provider generates the highest utility, however, is not statistically significant. The negative estimate of the no-choice option ($\widehat{\beta }$ -1.4499; SE 0.1198) indicates that respondents derived negative utility from not choosing one of the two tools offered. It can, hence, be concluded that radiologists are in general interested in AI-based assistance tools. The price estimate ($\widehat{\beta }$-0.1607; SE 0.1198) indicates typical cost-averse preferences. The provider attribute is not statistically significant (p 0.1404) implying it did not significantly impact the radiologists’ choice. The application type, on the other hand, was of significant interest (p < 0.0001) with the routine diagnostics option being preferred ($\widehat{\beta }$0.2984; SE 0.0622) and the scan time reduction tool being the least preferred option ($\widehat{\beta }$-0.3896; SE 0.0579). According to expectations, higher quality tools ($\widehat{\beta }$0.2421; SE 0.039) and highest time savings ($\widehat{\beta }$0.3949; SE 0.0587) resulted in higher utility. When applying the conjoint-based approach of calculating relative importance weights, time savings represent the most important attribute (37.98%), followed by the application type (31.52%). Considering these results, radiologists cared more about the time savings than the actual application type itself or it’s quality impact when choosing between tools. This can also be observed regarding MWTP where time savings of 50% result in €2.46 MWTP and better quality only in €1.51 MWTP.

**Table 4:** Simple model utility estimates, marginal willingness to pay and relative importance of attribute levels (*n* = 114)

Attribute & attribute levels	p-value	Marginal utility$\widehat{\varvec{\beta }}$	Marginal WTP	Relative importance
**Provider**	0.1404			8.32%
Modality manufacturer		-0.05853	-0.36 €	
RIS/PACS software provider		-0.06151	-0.38 €	
AI-software startup		0.12004	0.75 €	
**Application**	< 0.0001			31.52%
Routine diagnostics		0.29843	1.86 €	
Process efficiency		-0.38958	-2.42 €	
Screening		0.09115	-0.57 €	
**Quality**	< 0.0001			22.18%
Same		-0.24206	-1.51 €	
Better		0.24206	1.51 €	
**Time Savings**	< 0.0001			37.98%
Low		-0.43392	-2.70 €	
Medium		0.03899	0.24 €	
High		0.39493	2.46 €	

**Table 5:** Conditional logit estimations for simple model (model I) and models incorporating subject-related interactions (models II-IV)

	*n* = 114	Model I (simple model)		Model II		Model III		Model IV	
	Variable	$\hat{\boldsymbol{\beta}}$ ***(SE)***	***p-value (LogW)***	$\hat{\boldsymbol{\beta}}$ ***(SE)***	***p-value (LogW)***	$\hat{\boldsymbol{\beta}}$ ***(SE)***	***p-value (LogW)***	$\hat{\boldsymbol{\beta}}$ ***(SE)***	***p-value (LogW)***
**Provider**			0.1404 (0.853)		0.1354 (0.868)		0.1275 (0.894)		0.1398 (0.855)
	L1: Modality manufacturer	-0.0585 (0.0603)		-0.0569 (0.0607)		-0.0589 (0.0608)		-0.0573 (0.0613)	
	L2: RIS/PACS provider	-0.0615 (0.0631)		-0.0652 (0.0636)		-0.0653 (0.0637)		-0.0647 (0.0641)	
	L3: AI-software startup	0.12 (0.0608)		0.1221 (0.0613)		0.1242 (0.0614)		0.122 (0.0617)	
**Application**			<0.0001 (11.578)		0.0013 (2.889)		0.0014 (2.852)		<0.0001 (4.586)
	L1: Diagnostics (routine diagnostics)	0.2984 (0.0622)		0.2909 (0.2433)		0.2886 (0.2387)		0.1655 (0.2519)	
	L2: Process efficiency (scan time reduction)	-0.3896 (0.0579)		-0.7046 (0.2504)		-0.6696 (0.238)		-0.8704 (0.2511)	
	L3: Screening support (mammography)	0.0911 (0.0642)		0.4136 (0.2434)		0.381 (0.2413)		0.7049 (0.254)	
**Quality**			<0.0001 (9.727)		0.0524 (1.281)		0.0521 (1.283)		0.0519 (1.285)
	L1: Same	-0.2421 (0.039)		-0.3084 (0.1583)		-0.3033 (0.153)		-0.3032 (0.1531)	
	L2: Better	0.2421 (0.039)		0.3084 (0.1583)		0.3033 (0.153)		0.3032 (0.1531)	
**Time savings**			<0.0001 (13.702)		<0.0001 (14.064)		<0.0001 (14.127)		<0.0001 (14.449)
	L1: Low	-0.4339 (0.0637)		-0.4414 (0.0643)		-0.4427 (0.0644)		-0.451 (0.0649)	
	L2: Medium	0.039 (0.0584)		0.0382 (0.0589)		0.0382 (0.059)		0.0398 (0.0594)	
	L3: High	0.3949 (0.0587)		0.4031 (0.0592)		0.4045 (0.0593)		0.4113 (0.0598)	
**Price**	Price per study	-0.1607 (0.0176)	<0.0001 (20.895)	-0.1634 (0.0178)	<0.0001 (21.275)	-0.1593 (0.0178)	<0.0001 (21.185)	-0.1618 (0.018)	<0.0001 (20.554)
**No-choice**	No-choice	-1.4499 (0.1198)	<0.0001 (33.422)	-1.4611 (0.1209)	<0.0001 (33.361)	-1.4762 (0.1214)	<0.0001 (33.885)	-1.4851 (0.1222)	<0.0001 (33.926)
**Subject-related interactions**	Gender[M]* Application[Diagnostics]			-0.0899 (0.246)	0.0014 (2.862)	-0.0903 (0.2414)	0.0014 (2.850)	-0.0783 (0.2417)	0.001 (3.009)
	Gender[M]* Application[Process]			0.3435 (0.2523)		0.306 (0.2402)		0.3241 (0.2403)	
	Gender[M]* Application[Screening]			-0.2536 (0.2453)		-0.2157 (0.2434)		-0.2458 (0.244)	
	Gender[F]* Quality[Better]			0.1605 (0.1663)	0.0161 (1.793)	0.1571 (0.1611)	0.021 (1.679)	0.1585 (0.1613)	0.0205 (1.689)
	Budget responsibility[Y]* Price					-0.0347 (0.0105)	0.001 (3.005)	-0.0366 (0.0106)	0.0005 (3.278)
	Specialization[Mammography]*Application [Screening]							0.3873 (0.0932)	<0.0001 (4.068)
**Model fit**	AICc	2197.69		2186.10		2177.31		2162.70	
	BIC	2242.88		2261.25		2257.44		2252.79	
	-2LogLikelihood	2179.53		2155.67		2144.82		2126.10	
	LogLikelihood	-1089.77		-1077.84		-1072.41		-1063.05	

*SE* Standard Error, *LogW *LogWorth, *AICc* Corrected Akaike Information Criterion, *BIC *Bayesian Information Criterion

### Adoption probability simulation

**Fig. 3:**
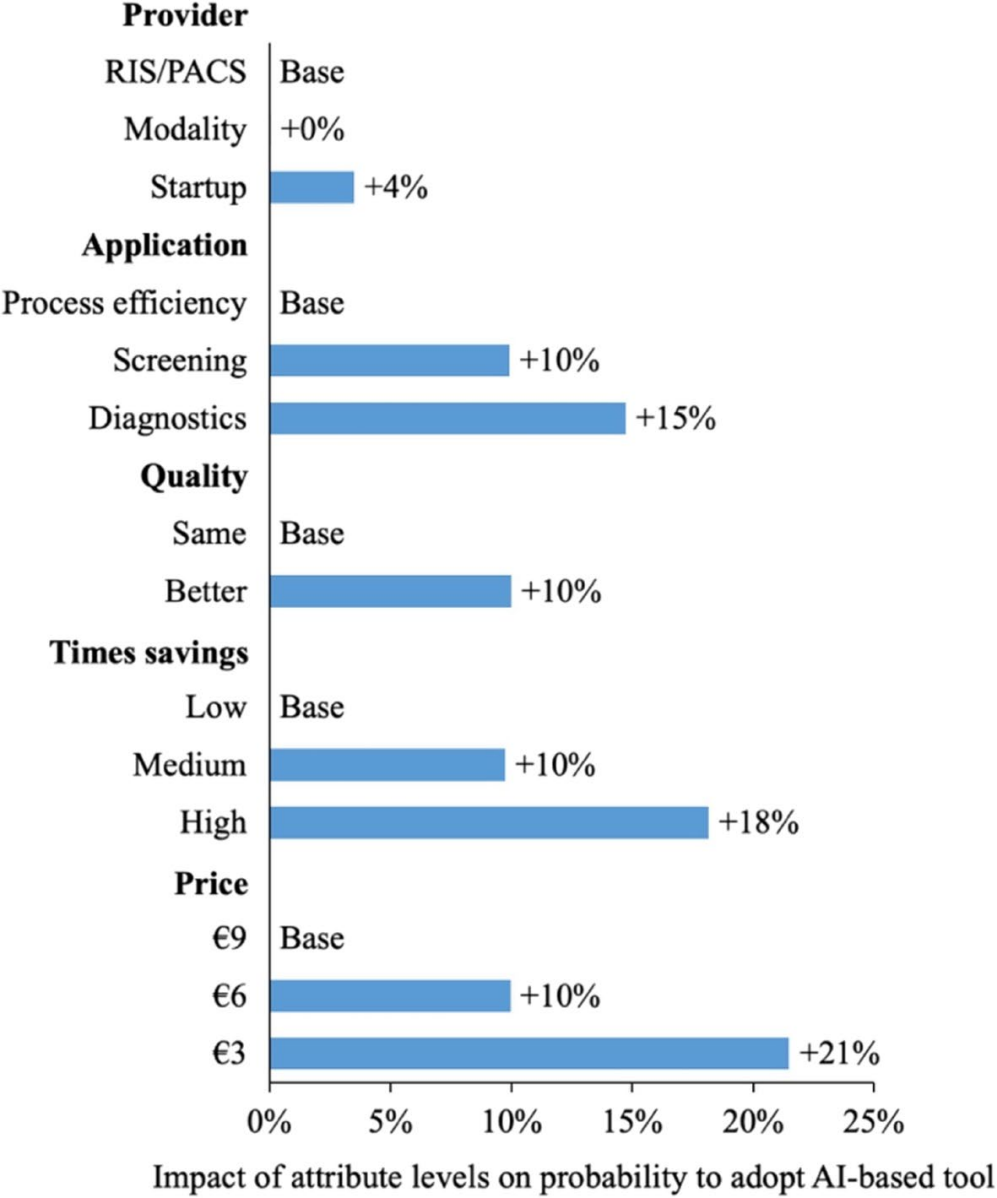
Effects of marginal changes in attribute levels on probability to adopt AI-based assistance tools

Applying Eq. 3, a general average tool adoption probability of $P\left(1\right)=\frac{1}{1 + exp(-1.4499)}=80.98\%$ was determined (95% CI 77.1% − 84.4%). To derive the impact on adoption probability of the single attribute levels, the least preferred combination was defined as a base case (B). The average utility derived from the base case can be calculated as ${U}_{B}=-0.061+\left(-0.3896\right)+\left(-0.2421\right)+\left(-0.4339\right)+\left(-0.1607*9\right)=-2.5736$. Building on Eq. 3 again, the probability of choosing the base case over the no-choice option was $P\left(B\right)=\frac{\text{e}\text{x}\text{p}(-2.5736)}{\text{e}\text{x}\text{p}(-2.5737)+exp(-1.4499)}=25\%$. To determine the individual impact on adoption probability of the single attribute levels, single levels were altered keeping all others constant. For example, reducing the price of the base case to €3 implies an adoption probability of $\frac{exp(-1.6093)}{exp(-1.6093)+exp(-1.4499)}=46\%$, hence an increase of 21%. Figure 3 visualizes how these improvements to the most preferred levels impact on adoption probability. The highest impact can be identified for time savings and price here.

### Subject-related interaction effects

Models II to IV of Table 5 incrementally add subject-related interaction effects to the simple model. Considering model II, gender shows significant interaction effects with the application and quality attributes. On average, male radiologists prefer the process efficiency application (i.e., scan time reduction) more than women or diverse respondents (p 0.0014; $\widehat{\beta }$0.3435 SE 0.2523). Women, derive a higher utility from a tool that provides better-than-human quality than men or diverse respondents (p 0.0161; $\widehat{\beta }$0.1605 SE 0.1663). The estimation of model III introduces the relationship between financial responsibility of respondents and WTP. In this case, chief physicians in hospitals and self-employed practice owners were defined as budget responsible since these individuals are factually also responsible for the financial performance in Germany. Model III implies that respondents with budget responsibility are more cost-averse deriving an even lower utility from the price estimate (p 0.001; $\widehat{\beta }$-0.0347 SE 0.0105). It is, however, important to make the reader aware that factual “budget responsibility” does not necessarily imply “budget awareness” or “financial skills”. Theoretically, some of the respondents defined as responsible for the budget might not have considered economic aspects when completing the experiment, even though these results imply the opposite. Lastly, and somehow intuitively, respondents that were specialized in mamma diagnostics and screening also preferred the screening application (p < 0.0001; $\widehat{\beta }$0.3873 SE 0.0932). The model fit consistently improved from model I to IV as indicated by decreasing AICc, BIC and − 2LogLikelihood values.

At this point it is also important to mention tested interaction effects that did not generate statistically significant results. These include age, specialization, perceived level of knowledge regarding AI in general and in radiology specifically, trust in AI in radiology, level of technophilia and the opinion on whether AI in radiology can save costs or increase efficiency. Moreover, no statistically significant differences could be observed when differentiating between users and non-users of AI in radiology, as well as, between the inpatient/hospital and outpatient/practice sectors.

## Discussion

### General discussion

The described results provide concrete answers to the research question of this work. Firstly, radiologists are willing to invest in AI-based assistance tools as presented in the DCE indicated by an average 81% investment probability. Secondly, the most desired design of a tool was also identified. On average, radiologists preferred a tool that assists in routine diagnostics which performs at above-radiologist-level quality and saves 50% in diagnostics time at a price-point of €3 per study. The provider type of the respective tool did not appear to be a relevant factor for investment decisions. This fact can be considered good news for unestablished startup companies competing with the established modality and software players. Keeping an industry perspective, the current predominant development trajectory of diagnostics-related applications seems to meet radiologists’ preferences. In terms of pricing, radiologists are in general rather cost-averse with an increase in price from €3 to €9 decreasing the investment probability by 21%. A look at attribute importance generates insights, as well. More than for the application itself or its quality impact, radiologists care for the time it saves them. Considering that the average radiologist in the sample creates 43 reports per day, this appears comprehensible. Similarly, radiology is considered as one of the most capital intense medical specialties with high investments for equipment. Radiologists could therefore strongly consider the economics of their operations. Significant time savings can ultimately lead to an increased patient capacity, equipment utilization and, hence, amortization. An economically thinking profession would ultimately attach the highest importance to an attribute related to efficiency. Interestingly, female radiologists derive a higher utility from an improved quality than their male colleagues. One can only speculate whether this could be related to a more risk-averse mindset among female radiologists. Previous research has shown that female physicians are in general more likely to adhere to clinical evidence-based guidelines [45]. An assistance tool could simplify and automate this adherence resulting in higher quality care. In contrast to the expectations of most of the consulted experts, an application that reduces scan times and thereby improves process efficiency was the least preferred application type. In theory, reduced scan times also imply a higher equipment capacity and thereby additional economic benefits. However, the results imply that radiologists are primarily interested in tools that are immediately related to their everyday work, i.e., diagnostics. Scan processes could be mentally further away since they are mostly executed by medical technical assistants. At this point it is important to also mention tested subject-related interaction effects that turned out not to be statistically significant. For example, preferences were not significantly different when accounting for the age of respondents. In line with existing research, one would assume younger radiologists to be more open to new technologies like AI. A sample bias seems unlikely, since the average age of 51 years in this study is very much in line with the average age of the research population (52,4 years) [40]. Nevertheless, a possible goal for future research could be to capture a larger share of younger radiologists still in training. Secondly, the knowledge level regarding AI in radiology and trust in AI in radiology did also not significantly impact preferences. One would assume that radiologists who are more informed about AI or put high trust in AI would be willing to show a higher adoption probability or even a higher WTP. Thirdly and maybe most surprisingly, no significant differences in preferences could be identified between the 46% users and 50% non-users of AI, as well as, between the 25% of inpatient/hospital and 75% outpatient/practice radiologists. Considering AI usage, this could indicate that the hopes and ideas regarding AI of current non-users very much mirror the actual experience of users. For example, non-users did not choose the “no-choice” option significantly more often than users and vice versa. Keeping in mind the relatively high adoption probability of 81%, hopes and ideas and actual experience would then most likely be of positive nature. When looking at differences between the inpatient and outpatient sector, one must consider the significant bias towards outpatient respondents in the study sample. These working environments do differ in practice and reimbursement mechanisms. Directly related to this is also the degree of specialization which tends to be higher in hospitals [40]. Future research considering a more balanced study sample could, hence, identify differences. In the end, the results also provide important information for policymakers attempting to drive future adoption of quality-enhancing AI-based tools in radiology. The past has shown that healthcare providers are oftentimes not willing to invest in digital solutions. Until 2017, for example, more than half of German hospitals did not have an electronic health record installed and still relied on paper-based documentation [46]. The lawmaker ultimately reacted via the 2020 Hospital Future Law which also introduces sanctions for not installing several digital tools starting from 2025 [47]. This study, however, shows that radiologists are likely to voluntarily invest and that this is especially the case for tools promising significant efficiency gains. Policymakers are highly interested in improving quality of care via innovative technology like AI. When setting up governmental funding programs for the development of new technology, however, also properties like efficiency improvements should be considered. This ultimately increases the probability that funded technology is also adopted by providers like radiologists without the need for controversial instruments like sanctions. Similarly, most partly publicly funded research currently still focuses on proving the sensitivity or specificity of AI-based applications, which is a logical first step. In the future, however, researchers need to broaden the study spectrum to also include efficiency parameters keeping physicians’ preferences in mind. Ultimately, our results also provide insights for policymakers on a systemic level. As previously mentioned, scientific proof for quality-enhancing effects of AI in radiology, but also of digitization in healthcare overall, exists. Even though one must expect most practitioners to care about quality of care, the same practitioners also need a viable business case when investing in quality-enhancing technology. Hence, there needs to be a financial incentive either in the form of cutting costs, increasing productivity or additional revenue via higher reimbursement. It can be argued that additional revenue represents the most immediate and impactful incentive among these options. Productivity improvements, for example, are very much dependent on the specific circumstances in practices and hospitals. So far, the German healthcare system has not significantly pivoted towards value-based reimbursement that rewards improvements in care quality [48]. In the outpatient sector, reimbursement is strictly provided by volume. In the hospital sector, the introduction of Diagnosis Related Groups (DRGs) in 2003 represented a limited move towards considering outcomes in reimbursement schemes, e.g., via financial penalties for early readmissions [49]. In summary, the financial rewards, in terms of reimbursement for increasing care quality are very limited. It can be assumed that the results of this work might be impacted by this underlying reimbursement system. In terms of pricing, radiologists were comparatively cost-averse with an increase in price reducing the adoption probability more than any other attribute level change. Additionally, time savings were considered more important than an increase in quality. Finally, one could hypothesize that a significant shift towards value-based healthcare systems and, hence, quality-based reimbursement also increases the relative perceived importance of quality or even the adoption of quality-enhancing technology in healthcare.

### Limitations

The previously discussed results also face potential limitations. First and foremost, the creation of a contingent market including the choice of attributes and attribute levels always implies simplification. Attributes not included in the DCE could have been of interest to radiologists. This especially applies to the application attribute considering the variety of AI-based solution currently under development. The authors, however, tried to address this limitation by defining three options that represent three archetypes of tools (routine diagnostics, process efficiency & screening). Moreover, the expert interviews helped to correctly frame the application levels and to assure a similar attractiveness avoiding level dominance. On a similar note, differently phrased levels for the quality attribute were used depending on the application displayed. An improvement in image quality might not have the same perceived quality impact as an improvement in diagnostics quality for some respondents. One last potential limitation related to the contingent market is the choice of price levels. Radiologists are currently rarely confronted with fees on a per-study basis since equipment and software providers usually negotiate long-term service contracts. Hence, respondents likely did not have other prices for comparison in mind. This could have resulted in an anchor effect related to the chosen price points distorting actual WTP. Nevertheless, the authors tried to minimize this limitation via expert interviews and by linking the prices to reimbursement. Besides limitations related to the contingent market, the sample should be considered. Firstly, only the preferences of German radiologists are mirrored here. Despite partnerships with the major professional associations of German radiologists, only 119 radiologists completed the DCE. This clearly has a potential for bias in our results. Nevertheless, for several reasons this bias can be considered limited. Firstly, the study sample shares several similarities with the underlying sample frame as presented in Supplementary Table 3 of Additional File 1 except for the practice-hospital split. Specifically, our sample represents a similar average age, gender split and specialization structure when compared with publicly available statistics on all German radiologists. Secondly, the final sample exceeds the identified minimum required sample size of 75 enabling the estimation of a statistically reliable model. Thirdly, in terms of sample size, this study is in line with a large share of other DCEs in healthcare. For example, de Bekker-Grob, Donkers [50] show that reviewed DCE-based studies in healthcare rely on a sample size of less than 300 respondents in 50% and less than 100 in 25% of the cases. At the same time, it is important to consider that most of these studies are patient-focused with underlying sample frames of several million individuals. This study was confronted with a comparably small sample frame of 9,313 fully trained radiologists in Germany. More importantly to the generalizability of results, the sample was skewed towards respondents working in the outpatient sector (74%) potentially limiting validity of results for the hospital sector. As mentioned in the Discussion, work environments and reimbursement do significantly differ between these sectors. We did not identify statistically significant differences between them, which could have been the case with a more balanced sample. Since hospitals also have a higher degree of specialized radiologists, the same holds for differences between specialized and non-specialized respondents which were not detected in this study. Finally, the sampling strategy of collecting responses via mailing lists can result in a biased final sample that is more open for the topic than the average German radiologist.

## Conclusions

This study suggests that physicians, here exemplified by German radiologists, are overall willing to invest in AI-based assistance tools. This applies to both current users and non-users of AI-based tools with no significant difference. They prefer applications that immediately support everyday tasks like routine diagnostics or diagnostic screening over applications that are focused on process efficiency via scan time reductions. The provider type of the tool is of no significant interest in the choice process, hence leveling the playing field between established equipment or software providers and uprising startups. The most important feature when choosing a tool appears to be its potential to save time. This feature is even considered more important than quality improvements (e.g., detecting anomalies at above-human-level performance). AI-based assistance tools do have the potential to improve the quality of our healthcare. This work shows, however, that tool development and funding should always consider the features of immediate everyday and economic relevance for the physicians like time savings.

## Supplementary Information

**Additional file 1.**

**Additional file 2.**

## Data Availability

The datasets generated and/or analyzed during the current study are available from the corresponding author on reasonable request.

## Abbreviations

2D/3D: Two/three-dimensional
AI: Artificial intelligence
AICc: Corrected Akaike information criterion
BIC: Breast imaging reporting and data system
CI: Confidence interval
CT: Computed tomography
DCE: Discrete choice experiment
DL: Deep learning
DRG: Diagnosis Related Group
ML: Machine learning
MRI: Magnetic resonance imaging
MWTP: Marginal willingness to pay
PACS: Picture archiving & communication system
RIS: Radiology information system
SD: Standard deviation
SE: Standard error
WTP: Willingness to pay
