# Supplementary material for: Physicians’ preferences and willingness to pay for artificial intelligence-based assistance tools: a discrete choice experiment among german radiologists
Source: BMC Health Serv Res. 2022 Mar 26;22:398. doi: 10.1186/s12913-022-07769-x (PMC8959781; doi:10.1186/s12913-022-07769-x)
Supplement: Supplementary file 1 — Additional file 1. [file 12913_2022_7769_MOESM1_ESM.pdf]

Additional File 1

Supplement to: Physicians' Preferences and Willingness to Pay for Artificial Intelligence-based Assistance Tools: A Discrete Choice Experiment Among German Radiologists

Supplementary Table 1. Long-list of attributes and attribute levels and exclusion process to arrive at short list

	Attributes	Attribute levels
1	Provider	– Modality manufacturer<br>– RIS/PACS software provider<br>– AI-software startup<br>– *University hospital*
2	Application	– Automatic marking of lung lesions in thoracic CT and liver and kidney lesions in abdominal MRI<br>– Reduction of scan times for 2D & 3D abdominal MRI sequences via AI-based data manipulation<br>– Presorting of mammographic screening reports into "100% normal" (BI-RADS 1&2) and "suspicious" incl. automatic lesion marking<br>– *Automatic detection and marking of carcinoma of the prostate in MRI scans incl. contouring for potential biopsy*<br>– *Automatic morphometry in MRI brain scans incl. volumetry and lesion detection*<br>– *Probability of COVID-19 infection (in %) based on lung CT scan*
3	*Automatic differential diagnosis proposal?*	– *Yes*<br>– *No*
4	*Automatic report creation?*	– *Yes*<br>– *No*
5	Quality	– *Worse*<br>– Same<br>– Better
6	Time savings	– Low<br>– Medium<br>– High
7	Pricing	– €3 per study<br>– €6 per study<br>– €9 per study

Excluded attributes/levels

2/3D indicates two/three-dimensional; AI, Artificial Intelligence; BI-RADS, Breast Imaging Reporting and Data System; CT, Computed Tomography; MRI, Magnetic Resonance Imaging; PACS, Picture Archiving and Communication System; RIS, Radiology Information System

Supplementary Table 2. Experimental design and efficiency

Choice set	Attributes 1	2	3	4	5
1	2	2	1	2	6
	3	1	2	3	3
2	1	3	1	2	3
	2	2	2	1	9
3	1	2	1	1	3
	3	1	2	2	6
4	3	3	1	1	9
	1	2	2	3	6
5	3	2	1	3	3
	1	3	2	2	9
6	2	3	2	3	9
	3	1	1	1	6
7	1	1	1	3	9
	3	2	2	2	3
8	2	3	1	3	6
	1	2	2	2	9
9	2	1	2	1	3
	3	2	1	3	9
10	1	3	2	1	6
	2	1	1	2	3
D-efficiency: 92.35%					

Supplementary Table 3. Overview of sample frame characteristics

Sample & sample frame characteristics		% sample	% sample frame
Gender	Male	78%	66%
	Female	21%	33%
Age	Mean	51	52.4
Job position	Total outpatient	74%	48%
	Total inpatient	25%	46%
	Employed in public authority	1%	1%
	Other	0%	5%
Specializations	Pediatric radiology	1%	2%
	Neuroradiology	7%	7%

Source: Ärztestatistik zum 31. Dezember 2020, Bundesärztekammer (retreived from: https://www.bundesaerztekammer.de/fileadmin/user_upload/downloads/pdf-Ordner/Statistik_2020/2020-Statistik.pdf)

Supplementary Figure 1. The relationship between current AI usage and plans for (further) investments in AI

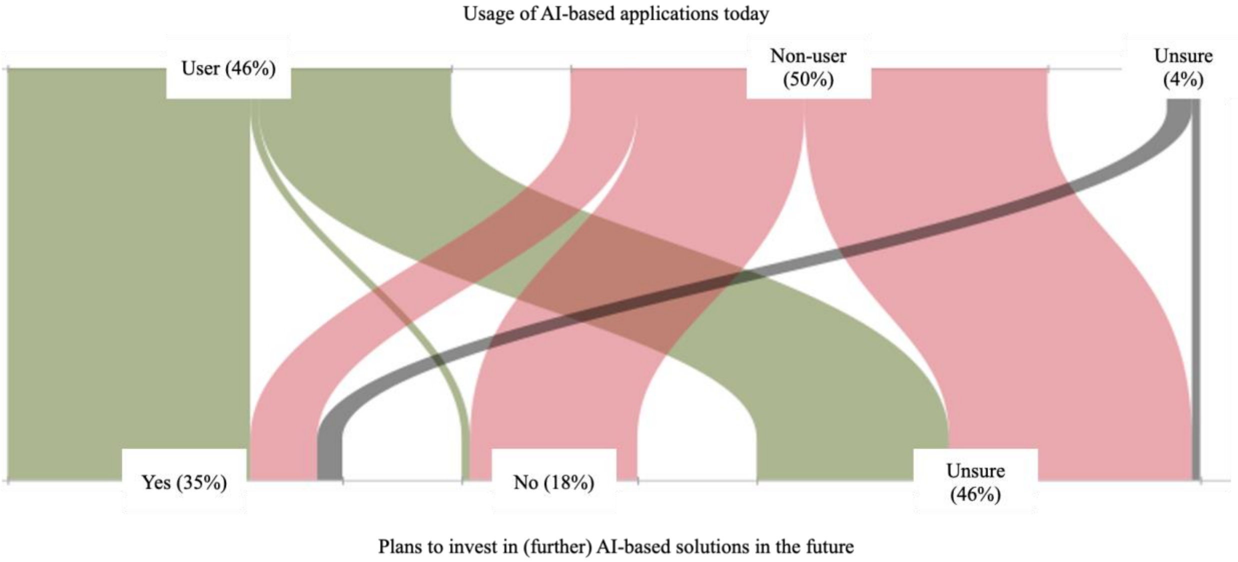

AI indicates Artificial Intelligence; Note: figures rounded
