# Supplementary material for: Physicians’ preferences and willingness to pay for artificial intelligence-based assistance tools: a discrete choice experiment among german radiologists
Source: BMC Health Serv Res. 2022 Mar 26;22:398. doi: 10.1186/s12913-022-07769-x (PMC8959781; doi:10.1186/s12913-022-07769-x)
Supplement: Supplementary file 2 — Additional file 2. [file 12913_2022_7769_MOESM2_ESM.pdf]

Survey instrument (translated from German)

1 Start

Survey on Artificial Intelligence (AI) in radiology

Dear participant,
Artificial Intelligence, or AI, slowly but steadily finds its place in our everyday lives, as well as, in healthcare. Especially in radiology quite a few successes were achieved in the last couple of years.

You, being a radiologist yourself knows best in which situations and how AI can best assist your daily work. As a matter of fact, in the near future you will most likely be able to choose from a variety of AI-based assistance tools.

Interestingly, physicians' preferences are rarely considered in public discussions. This survey attempts to change this and concerns the following question:

„How do radiologists perceive AI-based applications and which features are considered important when choosing such a tool?"

As an appreciation of your time we provide an overview of all currently available AI-based radiology applications in Germany. Furthermore, feel free to leave your mail address to receive information on the results of the study attached to this survey. Self evidently, all answers are treated confidentially and are not used for commercial purposes.

———

How is this survey structured?

This survey will take approx. 10-15 min. of your time and is composed of 2 parts:

1. Questions regarding you as a person, your work environment & existing experience with AI
2. Task to determine your preferences regarding AI-based assistance tools in radiology

Who is conducting this survey?

This research study is conducted by BLINDED. In case of questions or comments, feel free to reach out via mail:
BLINDED

Thank you very much for your time!

2 Part 1: General questions

Gender

- ◯ Female
- ◯ Male
- ◯ Diverse

Age

[ ]

Where do you practice your job

- ◯ Hospital
- ◯ University hospital
- ◯ Radiology practice
- ◯ Medical Services Center (MSC)
- ◯ Public authority
- ◯ Other [ ]

In which position do you practice your job?

- ○ Self-employed in practice
- ○ Chief physician in hospital
- ○ Consulting physician in hospital
- ○ Resident physician in hospital
- ○ Employed by practice/MSC
- ○ Employed by public authority
- ○ Retired

How many reports do you autonomously create per day (rough average)?

[ ]

How many reports do you validate per day (rough average)?

[ ]

Do you have a specialization?

Specialization here means, that more than 70% of your time is spent on this area.

- ○ No specialization covering more than 70% of my time
- ○ Neuroradiology
- ○ Pediatric radiology
- ○ Interventional radiology
- ○ Mamma (breast) diagnostics
- ○ Musculoskeletal diagnostics
- ○ Oncological diagnostics
- ○ Conventional radiology (x-ray)
- ○ Other [ ]

How do you assess the following statements regarding digitalization?

	Strongly agree	Agree	Neither	Disagree	Strongly disagree
I am technophile in my private life.	○	○	○	○	○
I keep myself regularly informed about technical novelties regarding my job.	○	○	○	○	○
My work environment and respective processes are digitalized.	○	○	○	○	○

The amount and variety of digital applications in radiology is increasingly difficult to grasp.	○	○	○	○	○

3 Part 1: AI-related questions

How many years of work experience as a fully trained radiologist do you have?

- ○ 1-3
- ○ 4-6
- ○ 7-9
- ○ 10-12
- ○ 13+

In which year of your residenacy are you currently?

- ○ 1-2
- ○ 3-4
- ○ 5-6
- ○ 6+
- ○ No residency in radiology

How do you assess the following statements regarding AI?

	Strongly agree	Agree	Neither	Disagree	Strongly disagree
I follow the public discussion regarding AI.	○	○	○	○	○
AI will replace radiologists in the future.	○	○	○	○	○
AI complements radiologists in the future.	○	○	○	○	○
AI can improve quality in radiology.	○	○	○	○	○
AI can improve efficiency in radiology.	○	○	○	○	○
AI can save costs in radiology.	○	○	○	○	○
I trust AI in radiology.	○	○	○	○	○
I see the need for further education on AI in radiology.	○	○	○	○	○

In 5 years, patients will prefer radiologic institutions that additionally rely in AI-based support.	○	○	○	○	○
In 10 years, patients will prefer radiologic institutions that additionally rely in AI-based support.	○	○	○	○	○

How do you assess the following statements regarding the AI-based offering in radiology?

	Strongly agree	Agree	Neither	Disagree	Strongly disagree
We increasingly receive offers for AI-based applications from various providers.	○	○	○	○	○
We increasingly ask for offers for AI-based applications ourselves.	○	○	○	○	○
The pricing for AI in radiology is appropriate.	○	○	○	○	○
I feel well informed regarding the commercially available AI-based applications in radiology.	○	○	○	○	○
We ourselves develop AI to support diagnostics or improve process efficiency.	○	○	○	○	○
We ourselves develop AI because of our research agenda.	○	○	○	○	○

Do you currently utilize AI-based applications as a radiologist?

- ○ Yes
- ○ No
- ○ Unsure

Which features of AI-based assistance tools are most important for you?

Verschieben Sie die blauen Boxen von links nach rechts in Ihre gewählte Reihenfolge. Die für Sie wichtigste Eigenschaft steht auf der rechten Seite an oberster Stelle. (Auf kleinen Bildschirmen, z.B. Smartphones, bilden Sie eine Reihenfolge durch klicken auf die blauen Boxen; "1" entspricht hier der für Sie wichtigsten Eigenschaft.)

	1	2	3	4	5	6	7
The application saves time in diagnostics.	○	○	○	○	○	○	○
The application shortens scan times.	○	○	○	○	○	○	○
The application improves scan image quality.	○	○	○	○	○	○	○
The application saves costs.	○	○	○	○	○	○	○
I get reimbursed for using the application.	○	○	○	○	○	○	○
The application diagnoses better than human radiologists.	○	○	○	○	○	○	○
The application is approved as a medical device.	○	○	○	○	○	○	○

4 Part 1: AI usage questions

You indicated that your are currently using AI. In which areas is this the case?

- ☐ Supporting/speeding up diagnostics
- ☐ Prognosis of disease progression
- ☐ Creation of reports
- ☐ Improvement of image quality
- ☐ Shortening of scan times
- ☐ Replacing/reducing contrast agents
- ☐ Practice/ward process management
- ☐ Other ____________

Which providers of AI-based applications do you utilize?

- [ ] Philips
- [ ] Siemens Healthineers
- [ ] GE
- [ ] Other modality/equipment manufacturer
- [ ] RIS/PACS-software provider
- [ ] AI-software startup
- [ ] Own development
- [ ] Other ______
- [ ] Unsure

Do you plan to invest in (further) AI-based assistance tools in the next 2 years?

- ( ) Yes
- ( ) No
- ( ) Unsure

5 Part 2: Introduction

Please imagine the following scenario:

Several providers are offering different AI-based assistance tools. The offers differ regarding their general application type, other parameters and in price. Now, you want to choose the best offer for your *practice/department.*

On the following 10 pages you have the choice between 2 differing tool options. Furthermore, you can always decide against adopting one of the two displayed offers.

IMPORTANT: Please imaging that there are no alternative tool options than the ones displayed on each of the 10 pages. Please assume that you are not able to adopt options you did not choose any other way, even if you are currently using a similar tool in your practice/department. It is of utmost importance for this study that you complete all 10 choice sets.

6 Part 2: DCE CS 1

Choice 1/10

	Offer 1	Offer 2
Provider:	RIS/PACS software provider	AI software startup
Application:	Reduction of scan times for 2D & 3D abdominal MRI sequences via AI-based data manipulation	Automatic marking of lung lesions in thoracic CT and liver and kidney lesions in abdominal MRI
Quality:	Same: Same image quality	Better: Detects anomalies you would not detect even with long inspection
Time savings:	Medium: MRI scan process 30% faster	High: Diagnostics process 50% faster
Price	6 € per study	3 € per study

Which offer do you prefer?

- Offer 1
- Offer 2
- None of these

Interpretation (if needed):
Offer 1 comprises an assistance tool provided by a RIS/PACS-software provider (e.g., AGFA/Dedalus, Medavis, etc.) which cuts scan times for MRI scans of the abdomen by 30% at same image quality. The application of the tool is priced at 6€ per study.
Offer 2 comprises an assistance tool provided by an AI-software startup which supports routine diagnostics in the mentioned areas (the markings are integrated in your existing PACS software). The system detects anomalies that you would not detect even after long examination and does this at a speed that cuts your diagnostics process time by 50%. The application is priced at 3€ per study.

7 Part 2: DCE CS 2

Choice 2/10

	Offer 1	Offer 2
Provider:	Modality provider	RIS/PACS software provider
Application:	Presorting of mammographic screening reports into “100% normal” (BI-RADS 1&2) and “suspicious” incl. automatic lesion marking	Reduction of scan times for 2D & 3D abdominal MRI sequences via AI-based data manipulation
Quality:	Same: Detects anomalies you would detect, too	Better: Higher image quality
Time savings:	Medium: Diagnostics process 30% faster	Low: MRI scan process 10% faster
Price:	3 € per study	9 € per study

Which offer do you pick?

- ○ Offer 1
- ○ Offer 2 None
- ○ of these

8 Part 2: DCE CS 3

Choice 3/10

	Offer 1	Offer 2
Provider:	Modality provider	AI software startup
Application:	Reduction of scan times for 2D & 3D abdominal MRI sequences via AI-based data manipulation	Automatic marking of lung lesions in thoracic CT and liver and kidney lesions in abdominal MRI
Quality:	Same: Same image quality	Better: Detects anomalies you would not detect even with long inspection
Time savings:	Low: MRI scan process 10% faster	Medium: Diagnostics process 30% faster
Price:	3 € per study	6 € per study

Which offer do you pick?

- Offer 1
- Offer 2 None
- of these

9 Part 2: DCE CS 4

Choice 4/10

	Offer 1	Offer 2
Provider:	AI software startup	Modality provider
Application:	Presorting of mammographic screening reports into “100% normal” (BI-RADS 1&2) and “suspicious” incl. automatic lesion marking	Reduction of scan times for 2D & 3D abdominal MRI sequences via AI-based data manipulation
Quality:	Same: Detects anomalies you would detect, too	Better: Higher image quality
Time savings:	Low: Diagnostics process 10% faster	High: MRI scan process 50% faster
Price:	9 € per study	6 € per study

Which offer do you pick?

- Offer 1
- Offer 2 None
- of these

10 Part 2: DCE CS 5

Choice 5/10

	Offer 1	Offer 2
Provider:	AI software startup	Modality provider
Application:	Reduction of scan times for 2D & 3D abdominal MRI sequences via AI-based data manipulation	Presorting of mammographic screening reports into “100% normal” (BI-RADS 1&2) and “suspicious” incl. automatic lesion marking
Quality:	Same: Same image quality	Better: Detects anomalies you would not detect even with long inspection
Time savings:	High: MRI scan process 50% faster	Medium: Diagnostics process 30% faster
Price:	3 € per study	9 € per study

Which offer do you pick?

- Offer 1
- Offer 2 None
- of these

11 Part 2: DCE CS 6

Choice 6/10

	Offer 1	Offer 2
Provider:	RIS/PACS-SoftwareProvider	AI software startup
Application:	Presorting of mammographic screening reports into “100% normal” (BI-RADS 1&2) and “suspicious” incl. automatic lesion marking	Automatic marking of lung lesions in thoracic CT and liver and kidney lesions in abdominal MRI
Quality:	Better: Detects anomalies you would not detect even with long inspection	Same: Detects anomalies you would detect, too
Time savings:	High: MRI scan process 50% faster	Low: Diagnostics process 10% faster
Price:	9 € per study	6 € per study

Which offer do you pick?

- Offer 1
- Offer 2 None
- of these

12 Part 2: DCE CS 7

Choice 7/10

	Offer 1	Offer 2
Provider:	Modality provider	AI software startup
Application:	Automatic marking of lung lesions in thoracic CT and liver and kidney lesions in abdominal MRI	Reduction of scan times for 2D & 3D abdominal MRI sequences via AI-based data manipulation
Quality:	Same: Detects anomalies you would detect, too	Better: Higher image quality
Time savings:	High: MRI scan process 50% faster	Medium: MRI scan process 30% faster
Price:	9 € per study	3 € per study

Which offer do you pick?

- Offer 1
- Offer 2 None
- of these

13 Part 2: DCE CS 8

Choice 8/10

	Offer 1	Offer 2
Provider:	RIS/PACS-SoftwareProvider	Modality provider
Application:	Presorting of mammographic screening reports into “100% normal” (BI-RADS 1&2) and “suspicious” incl. automatic lesion marking	Reduction of scan times for 2D & 3D abdominal MRI sequences via AI-based data manipulation
Quality:	Same: Detects anomalies you would detect, too	Better: Higher image quality
Time savings:	High: MRI scan process 50% faster	Medium: MRI scan process 30% faster
Price:	6 € per study	9 € per study

Which offer do you pick?

- Offer 1
- Offer 2 Noen
- of these

14 Part 2: DCE CS 9

Choice 9/10

	Offer 1	Offer 2
Provider:	RIS/PACS-SoftwareProvider	AI software startup
Application:	Automatic marking of lung lesions in thoracic CT and liver and kidney lesions in abdominal MRI	Reduction of scan times for 2D & 3D abdominal MRI sequences via AI-based data manipulation
Quality:	Better: Detects anomalies you would not detect even with long inspection	Same: Same image quality
Time savings:	Low: Diagnostics process 10% faster	High: MRI scan process 50% faster
Price:	3 € per study	9 € per study

Which offer do you pick?

- Offer 1
- Offer 2 None
- of these

15 Part 2: DCE CS 10

Choice 10/10

	Offer 1	Offer 2
Provider:	Modality provider	RIS/PACS-SoftwareProvider
Application:	Presorting of mammographic screening reports into “100% normal” (BI-RADS 1&2) and “suspicious” incl. automatic lesion marking	Automatic marking of lung lesions in thoracic CT and liver and kidney lesions in abdominal MRI
Quality:	Better: Detects anomalies you would not detect even with long inspection	Same: Detects anomalies you would detect, too
Time savings:	Low: Diagnostics process 10% faster	Medium: Diagnostics process 30% faster
Price:	6 € per study	3 € per study

Which offer do you pick?

- Offer 1
- Offer 2 None
- of these

16 Thank you

Dear participant,
thank you very much for your time. Your answers can provide valuable insights for future public health decisions and the development of actually preferred assistance tools.
As promised, you find an overview of currently available commercial AI-based assistance tools on the following page.

Please leave your mail address if you are interested in a results report.

To finish the survey, please click on "Next".

17 End

The survey was completed successfully. Click here for the promised AI-based tool overview. Feel free to reach out in case of questions and comments: BLINDED

Thank you very much for your participation!
